# Robust Brewed Tea Waste/Reduced Graphene Oxide Hydrogel for High Performance Flexible Supercapacitors

**DOI:** 10.3390/polym16223170

**Published:** 2024-11-14

**Authors:** Dan Wu, Jiajia Zhou, Wuqiang Deng, Guowen He, Zheng Liu

**Affiliations:** 1 College of Materials and Chemical Engineering, Hunan City University, Yiyang 413000, China; wudanwd@hnu.edu.cn (D.W.); 15616298559@163.com (J.Z.); 19958821050@163.com (W.D.); 2 Key Laboratory of Low Carbon and Environmental Functional Materials of College of Hunan Province, Yiyang 413000, China

**Keywords:** tea waste, reduced graphene oxide, hydrogel, supercapacitor

## Abstract

Tea waste contains various substances with phenolic hydroxyl groups, including lignin, tannins, tea polyphenols, etc., which are rarely utilized. In this study, tea waste was directly dispersed with graphene oxide to prepare tea waste/reduced graphene oxide (TW/rGO) hydrogel through a one-step hydrothermal method. The prepared hydrogel presented a continuous three-dimensional porous structure and exhibited good mechanical properties with a compressive strength of 53.4 ± 4.0 kPa. It also showed excellent electrochemical performance as an electrode material. Its specific capacitance reached 434.7 F g^−1^ at a current density of 1 A g^−1^, and its capacitance retention was 55.8% when the current density was increased to 100 A g^−1^. In addition, an TW/rGO assembled all-solid-state supercapacitor demonstrated a superior specific capacitance of 372.8 F g^−1^ and a competitive energy density of 12.9 Wh kg^−1^ at 1 A g^−1^.

## 1. Introduction

The growing environmental challenges and energy crisis, as well as the rapid development of portable/wearable electronics, have greatly stimulated the need for high-performance, green, and sustainable energy storage devices [1]. Supercapacitors store charges through the adsorption–desorption of ions at the electrode–electrolyte interface (electric double-layer capacitance) or pseudocapacitance based on rapid Faraday reactions [2]. With characteristics such as a high power density, long cycle life, and wide operating temperature range, supercapacitors have attracted widespread attention and undergone extensive development [3]. In particular, biomass-based supercapacitors, which are cost-effective, renewable, and environmentally friendly, are an appealing option for green and sustainable energy storage [4]. Various types of biomass, such as corn stalks [5], hazelnut shells [6], wheat straw [7], and tree bark [8], have been extensively used as renewable precursors for the fabrication of carbon-based electrodes in supercapacitor applications, owing to their varied compositions and morphologies, outstanding intrinsic structures, and renewability. However, biomass-derived carbons usually suffer from low capacitance and limited energy density due to their double-layer capacitance characteristics [3]. In contrast, biomass-derived redox-active biomolecules, such as juglone [9], rhein [10], polydopamine [11], and lignin [12,13], have demonstrated the ability to offer pseudocapacitance through reversible redox reactions, showing significant potential in the development of supercapacitors with high energy density. However, the acquisition of these biomass molecules involves complex extraction and purification processes [11,12]. Furthermore, their low electrical conductivity results in poor rate performance [10]. These factors limit their practical application in sustainable energy storage. Therefore, simple and efficient approaches are needed to convert biomass materials into valuable energy to achieve high specific capacitance and rate performance simultaneously.

Tea waste (TW), encompassing residues from both the tea making process and post-brewing, is a low-cost, high-yield, and biodegradable waste [14]. It is primarily subjected to landfilling or direct incineration, both of which impose significant burdens on environmental sustainability. Extensive research has been conducted on high-value-added treatments and the efficient use of TW to attain a satisfactory equilibrium between environmental conservation and economic benefits [14,15]. TW contains various bioactive compounds, such as proteins, polyphenols, minerals, lignin, cellulose, tannins, and so on [16], especially the abundant phenol groups existing in lignin and polyphenols, which can undergo a reversible redox reaction of quinone/hydrquinone and provide pseudocapacitance when adopted as electrodes for a supercapacitor [17,18]. Nevertheless, the redox-active biomolecules in TW are rarely utilized in a direct way. Additionally, the insulating nature of these biomolecules has posed a significant challenge for practical applications, as they adversely impact charge transport and storage capacity. Graphene, an ultra-thin two-dimensional (2D) nanomaterial characterized by its high specific surface area, exceptional electrical conductivity, and remarkable flexibility, is a good candidate for serving as a conductive skeleton for redox-active biomolecules. Jiang et al. employed industrial waste lignin as a reducing agent to convert graphene oxide into its reduced form via a hydrothermal reaction and obtained lignin-reduced graphene oxide (LRGO) aerogels [19]. These LRGO aerogels exhibited enhanced pseudocapacitive performance attributed to the reversible structural transition between phenol and quinone in lignin molecules. Xiong et al. reported a 3D porous tannin/grapheme (TAG) hydrogel created by combining tannin with graphene oxide through a hydrothermal process [20]. The as-prepared TAG achieved a high specific capacitance of 373.6 F g^−1^. However, there is a scarcity of research reports regarding the direct incorporation of TW with graphene for supercapacitor application.

In this study, TW was directly dispersed with graphene oxide (GO) to prepare tea waste/reduced graphene oxide (TW/rGO) hydrogel through a one-step hydrothermal method. The biomolecules in TW can interact with GO to form hydrogen bonds, π-π stacking, and chemical bonds. This enhanced interaction effectively alleviated the agglomeration of graphene sheets, and finally formed a robust 3D continuous porous network structure of TW/rGO hydrogel. Benefiting from the robust porous structure and existence of redox-active biomolecules, the as-prepared TW/rGO was capable of demonstrating outstanding specific capacitance and remarkable rate performance simultaneously.

## 2. Materials and Methods

### 2.1. Materials

Tea waste (TW) was derived from the residue of green tea after brewing. Natural graphite flake (325 mesh) was acquired from Henglide Graphite Co., Ltd. (Qingdao, China). Potassium permanganate (KMnO_4_), sodium nitrate (NaNO_3_), phosphoric acid (98% H_3_PO_4_), sulfuric acid (98% H_2_SO_4_), hydrochloric acid (37%, HCl), ammonia liquor (25% NH_3_·H_2_O), and hydrogen peroxide (30% H_2_O_2_) were obtained from Sinopharm Chem. Reagent Co., Ltd. (Shanghai, China) and utilized without further modification.

### 2.2. Preparation of Tea Waste/Reduced Graphene Oxide Hydrogel

Firstly, brewed green tea waste was refluxed with 5% dilute ammonia for 6 h, then filtered, dried, and ground to obtain TW powder. Graphene oxide (GO) was synthesized following the modified Hummers method, as detailed in our earlier research [21,22]. Secondly, the obtained tea waste powder was directly dispersed in 25 mL of GO solution (2 mg mL^−1^) containing 0.5 mL of H_3_PO_4_ (1 mol L) by stirring and ultrasonication. Finally, the above dispersion was placed into a Teflon-lined autoclave and subjected to hydrothermal treatment at 150 °C for 12 h, resulting in tea waste/reduced graphene oxide (TW/rGO) hydrogels. The mass ratios of GO/TW were 4:1, 6:1, and 8:1, and the corresponding hydrogels were labeled TW/rGO_1_, TW/rGO, and TW/rGO_2_, respectively. For comparative study, a pure rGO hydrogel was synthesized under identical conditions, excluding tea waste.

### 2.3. Fabrication of Electrode and Flexible Solid-State Supercapacitor

The working electrode in a three-electrode system was fabricated as follows. TW/rGO hydrogel, impregnated with a 1M H_2_SO_4_ aqueous solution, was sliced into 1 cm × 1 cm pieces, covered with a stainless steel mesh, and subsequently compressed at a pressure of 20 MPa to form an electrode. 

A flexible all-solid-state supercapacitor was fabricated as follows. Cellulose gel, prepared from microcrystalline cellulose as detailed in the Supplementary Materials, was utilized as both a separator and solid-state electrolyte. The electrode was fabricated by pressing one side of the TW/rGO hydrogel slice (1 cm × 1 cm) onto a stainless steel mesh collector under a pressure of 15 MPa. Next, two electrodes were stacked together with cellulose gel as a separator for assembly into an integrated device.

### 2.4. Electrochemical Measurements

Electrochemical performances were evaluated with a CHI760E electrochemical workstation (Shanghai Chenhua Instrument Co., Ltd., Shanghai, China). The three-electrode test involved immersing the platinum electrode (counter electrode), Hg/Hg_2_Cl_2_ electrode (reference electrode), and working electrode into 1 M H_2_SO_4_ electrolyte, followed by performing electrochemical impedance spectroscopy (EIS), galvanostatic charge–discharge (GCD), and cyclic voltammetry (CV) measurements. CV and GCD tests were conducted within a potential range of −0.2 to 0.8 V. EIS was conducted within a frequency range of 10^5^–0.01 Hz, employing an amplitude of 5 mV. The electrochemical performance of the flexible solid-state supercapacitor was evaluated through two-electrode measurements. The potential range for CV and GCD experiments spanned from 0 to 1 V. The test conditions for EIS were consistent with those of the three-electrode system.

The gravimetric specific capacitance (*C_m_*, F g^–1^) of the hydrogel electrode based on the three-electrode system was calculated using the following equation:

(1) $$C_{m}=\frac{I\times ∆t}{∆V\times m_{1}}$$

where I, ∆t, ∆V, and $m_{1}$ represent the charge–discharge current (A), discharge time (s), potential range (V), and the mass of one electrode (g), respectively.

For the flexible supercapacitor based on a two-electrode system, the specific capacitance (*C_s_*, F g^−1^), energy density (*E*, Wh kg^−1^), and power density (*P*, W kg^−1^) of supercapacitors were derived from the following equations, respectively:.

(2) $$C_{s}=\frac{I\times ∆t\times 4}{∆V\times m_{2}}$$

(3) $$E=\frac{C_{s}\times {∆V}^{2}}{8\times 3.6}$$

(4) $$P=\frac{E\times 3600}{∆t}$$

where $m_{2}$ represents the total mass of two electrodes (g).

### 2.5. Characterization

The morphology and structure of rGO and TW/rGO were characterized by SEM (SEM5000, Chinainstru & Quantumtech Co., Ltd., Hefei, China) and Raman microscopes (NOVA2S, Shanghai Ideaoptics Corp., Ltd., Shanghai, China), respectively. X-ray diffraction (XRD) measurements were conducted using a Smartlab SE X-ray powder diffractometer (Rigaku, Tokyo, Japan), employing Cu Kα radiation with a wavelength of λ = 0.154 nm. X-ray photoelectron spectroscopy (XPS) data were acquired using an AXIS SUPRA+ instrument (Shimadzu, Kyoto, Japan). The Brunauer–Emmett–Teller (BET) surface area was determined using a 3Flex surface analyzer (Micros Systems Inc., Huntington Beach, CA, USA) via N_2_ adsorption–desorption experiments. Samples for SEM, Raman, XRD, XPS, and BET tests were obtained by freeze-drying the corresponding hydrogels. Mechanical property data were acquired through compression tests conducted on hydrogel samples using an electronic universal testing machine (MTS E45.205, Mechanical Testing & Simulation, Ann Arbor, MI, USA).

## 3. Results and Discussion

**Figure 1:**
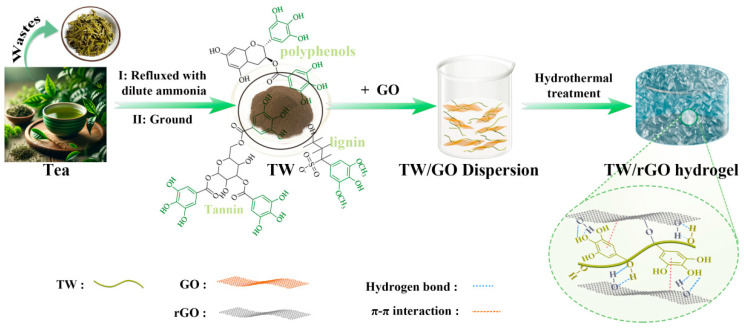
Schematic illustration of the preparation process for TW/rGO.

The fabrication process of TW/rGO hydrogel is schematically illustrated in Figure 1. Initially, the brewed green tea waste was treated with diluted ammonia to remove proteins and lipids, thereby enhancing its hydrophilicity. Next, the tea waste was ground to a powder and directly dispersed in a GO solution, followed by a hydrothermal treatment to form TW/rGO hydrogel. The functional groups within TW were capable of interacting with GO, resulting in a series of physical and chemical interactions, including hydrogen bonds, π bonds, and chemical bonds. These strong interactions allowed the TW to be firmly loaded on GO sheets, effectively preventing the aggregation of rGO sheets and cross-linking them, ultimately forming a 3D continuous porous network structure of TW/rGO hydrogel. The acquired TW/rGO hydrogel had a complete structure with a relatively rough surface (Figure S1). The volume of the TW/rGO hydrogel was larger than that of rGO, indicating a relatively loose structure with a lower degree of sheets aggregation in TW/rGO. In addition, TW/rGO hydrogel demonstrated outstanding mechanical strength, boasting a maximum compressive strength of 53.4 ± 4.0 kPa, significantly surpassing that of rGO (22.6 ± 2.9 kPa) (Figure S2). The enhanced mechanical strength of TW/rGO could be ascribed to the strong interactions between TW and rGO, which enhanced its structural stability and durability during charging and discharging processes [23].

**Figure 2:**
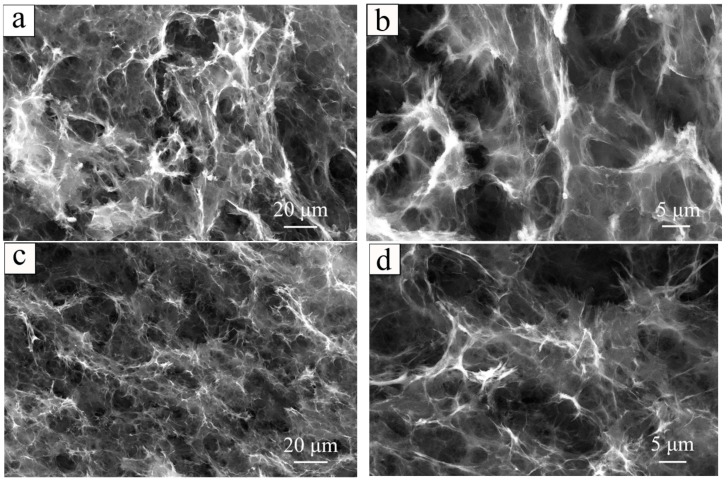
SEM images of (**a**,**b**) rGO and (**c**,**d**) TW/rGO.

Figure 2 presents scanning electron microscopy (SEM) images of rGO and TW/rGO. rGO showed a 3D interconnected porous framework featuring pore dimensions ranging from sub-micrometers to several tens of micrometers (Figure 2a). A portion of the aggregated graphene sheets could be observed in rGO (Figure 2b). In contrast, TW/rGO presented a similar morphology to that of rGO, but a more uniform and ordered porous structure, and no distinct aggregation of graphene sheets was observed (Figure 2c,d). These results indicate that the incorporation of TW in TW/rGO effectively alleviated the aggregation of graphene sheets. The uniform and ordered porous structure of TW/rGO can promote an efficient electron charge transfer within TW/rGO, thereby enhancing its electrochemical performance.

**Figure 3:**
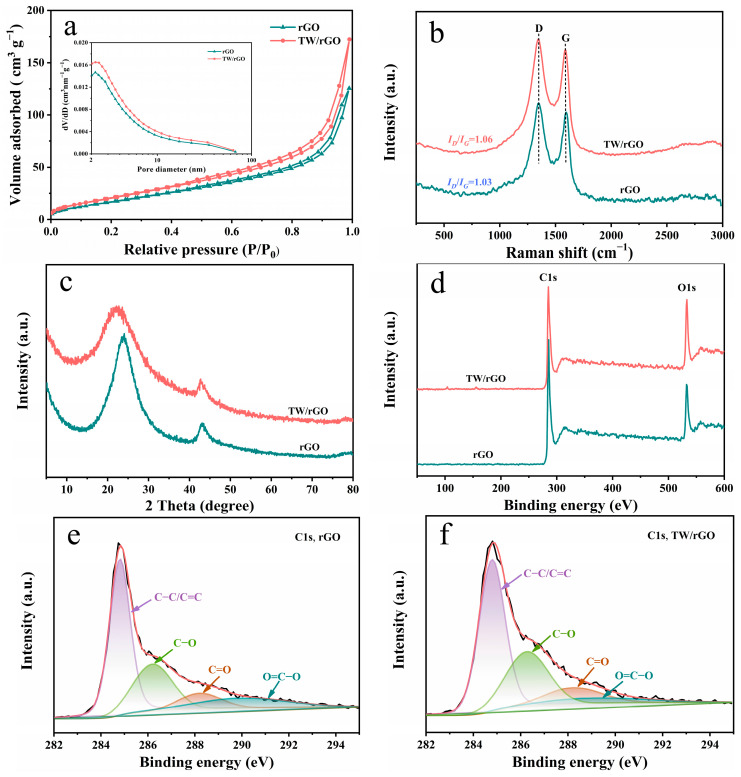
(**a**) Adsorption–desorption isotherm curves (the inset displays pore size distribution curves obtained using the Barrett–Joyner–Halenda method), (**b**) Raman spectra, (**c**) XRD patterns, (**d**) XPS survey, and C1s core-level spectra of (**e**) rGO and (**f**) TW/rGO.

The surface area and pore size distribution of the as-prepared porous hydrogels were investigated by BET N_2_ adsorption–desorption isotherms (Figure 3a). The specific surface area of TW/rGO was found to be 80.5 m^2^ g^−1^ (Table S1), which exceeds that of rGO (66.2 m^2^ g^−1^). In addition, as shown in Figure 3a, the N_2_ adsorption–desorption isotherm of TW/rGO exhibited a typical type-IV isotherm. The observation of an H3-type hysteresis loop, along with a significant rise in the high relative pressure range of P/P_0_ (0.9–1), indicates the presence of mesopores and macropores, respectively [24]. Meanwhile, the pore size distribution curve highlights the dominance of mesopores in TW/rGO, with a total pore volume of 0.27 cm^3^ g^−1^ and an average pore size of 13.3 nm. Dominant mesopores with a suitable pore size can ensure rapid ionic transport within the pore channel and increase the number of effective active sites, consequently enhancing energy storage performance [25]. 

Raman spectroscopy was carried out to reveal chemical structures. rGO and TW/rGO showed similar Raman patterns with two typical bands: D bands at ~1350 cm^−1^ and G bands at ~1590 cm^−1^ (Figure 3b). The D band corresponds to structural defects or partially disordered graphitic structures, while the G band is associated with the extensive graphitic domains in carbon materials. The intensity ratio of D/G bands (I_D_/I_G_) indicates the average distance between the structural defects. The I_D_/I_G_ of rGO and TW/rGO were measured to be 1.03 and 1.06, respectively. The slightly larger I_D_/I_G_ values of TW/rGO signify reduced graphitic domains and a rise in structural defects on rGO sheets, which probably originated from the chemical reaction between GO and TW.

X-ray diffraction (XRD) patterns were assessed to study the lattice structures of freeze-dried hydrogels, as shown in Figure 3c. For rGO, diffraction peaks at 2θ = 23.7° (d = 3.75 Å) and 43.2° (d = 2.09 Å) were assigned to the typical (002) and (100) planes of the graphite-like structure, respectively [26,27]. TW/rGO had a (002) peak shift to 2θ = 21.5° (d = 4.13 Å) with an increased half peak width compared to that of rGO (Table S2). The larger d-spacing was primarily attributed to strong interfacial interactions between TW and rGO, which slightly widened the gap between the stacked rGO sheets. These results indicate that TW was effectively incorporated into TW/rGO.

The elemental compositions of as-prepared samples were investigated via XPS. Figure 3d reveals that merely two peaks were identified at ~285 eV and ~533 eV in the XPS survey spectra for rGO and TW/rGO, which corresponded to C1s and O1s, respectively. The relative percentages of the elements are presented in Table S3. In comparison to rGO, the slightly increased oxygen content in TW/rGO was introduced by TW. The C1s core-level spectrum can be divided into four types of carbon groups (Figure 3e,f): C–C/C=C (284.8 eV), C–O (286.2 eV), C=O (288.2 eV), and O–C=O (290.0 eV). The relative content of C–O/C=O in TW/rGO was higher than that in rGO (Table S4), which was ascribed to the plentiful oxygen-containing functional group in TW. Although the relatively lower O–C=O content of TW/rGO might have been due to the presence of TW, it facilitated the elimination of oxygen-containing functional groups on GO [28]. These results provide additional confirmation of the successful preparation of TW/rGO.

**Figure 4:**
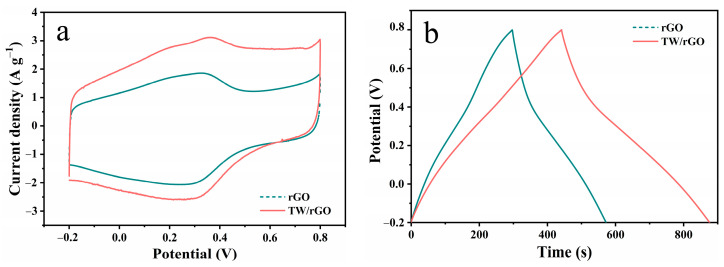

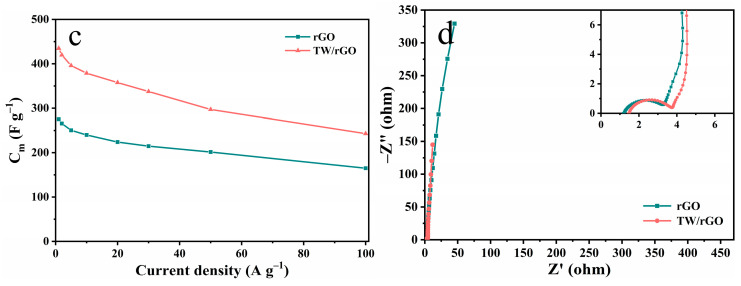
Electrochemical performances of rGO and TW/rGO hydrogels: (**a**) CV curves at a scan rate of 5 mV s^−1^, (**b**) GCD profiles at a current density of 1 A g^−1^, (**c**) plots of C_m_ versus current density, and (**d**) Nyquist plots.

The electrochemical performances of rGO and TW/rGO hydrogels were analyzed using a three-electrode configuration (Figure 4). As illustrated in Figure 4a, the CV curve of rGO presents a rectangular shape with distinct redox peaks at 0~0.4 V, denoting a combination of electrical double layer and pseudo-capacitive behaviors. The pseudocapacitance in rGO originated from the residual oxygen-containing functional groups after GO reduction, as shown in Equations (5)–(7) [29,30]. Compared to rGO, TW/rGO demonstrated a similar CV shape, but with a significantly enlarged area suggesting an enhanced charge-storage capability of TW/rGO. This was mainly due to the introduction of TW in TW/rGO, which brought an abundance of biomolecules with phenol groups. These groups can undergo reversible hydroquinone interconversion reactions, thereby providing additional pseudocapacitance, as shown in Equation (8) [17]. The redox reactions furnishing pseudocapacitance are depicted as follows:

(5) $$C-OH⇌C=O+H^{+}+e^{-}$$

(6) $$-COOH⇌-{COO}^{-}+H^{+}+e^{-}$$

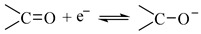
(7)

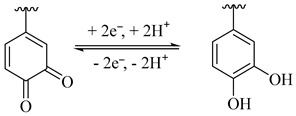
(8)

The obvious distortion from ideal triangular shapes in GCD curves of rGO and TW/rGO also validates the presence of pseudo-capacitances (Figure 4b). The specific capacitances (C_m_) of the hydrogels, as calculated from GCD curves, are depicted in Figure 4c. At 1 A g^−1^, the C_m_ of rGO and TW/rGO were 275.2 F g^−1^ and 434.7 F g^−1^, respectively. It should be noted that the capacitance of TW/rGO was 1.6 times higher than that of rGO, fully illustrating the significant capacitance contribution of TW. Furthermore, the capacitance of TW/rGO remained 243 F g^−1^ at the ultra-high current density of 100 A g^−1^, approximately 56% of that at 1 A g^−1^, demonstrating the remarkable rate performance of TW/rGO. The capacitance and rate performance of TW/rGO were exceptional compared to the majority of electrode materials derived from biomass (Table S5). Figure 4d displays the Nyquist plots of rGO and TW/rGO. In the high frequency region, small X-axis intercepts can be observed for both rGO and TW/rGO, suggesting a low equivalent series resistance (Rs) of the electrodes [31]. The diameter of the semicircle represents charge transfer and mass transfer resistance (Rct) [32]. In contrast, TW/rGO exhibited a slightly larger semicircle diameter, indicating a higher Rct. This was primarily due to the non-conductive nature of TW, which reduced the conductivity of the corresponding electrode. In the low frequency region, TW/rGO exhibited a steeper slope, indicating its ideal capacitance behavior with a faster ion diffusion rate [33], and further highlighting the superiority of its structure. The electrochemical performance of TW/rGO with different hydrothermal temperatures and different mass ratios of GO/TW are presented in Figure S3 and Figure S4, respectively. It can be concluded that the prepared TW/rGO showed the best performance at a 6:1 GO/TW mass ratio.

**Figure 5:**
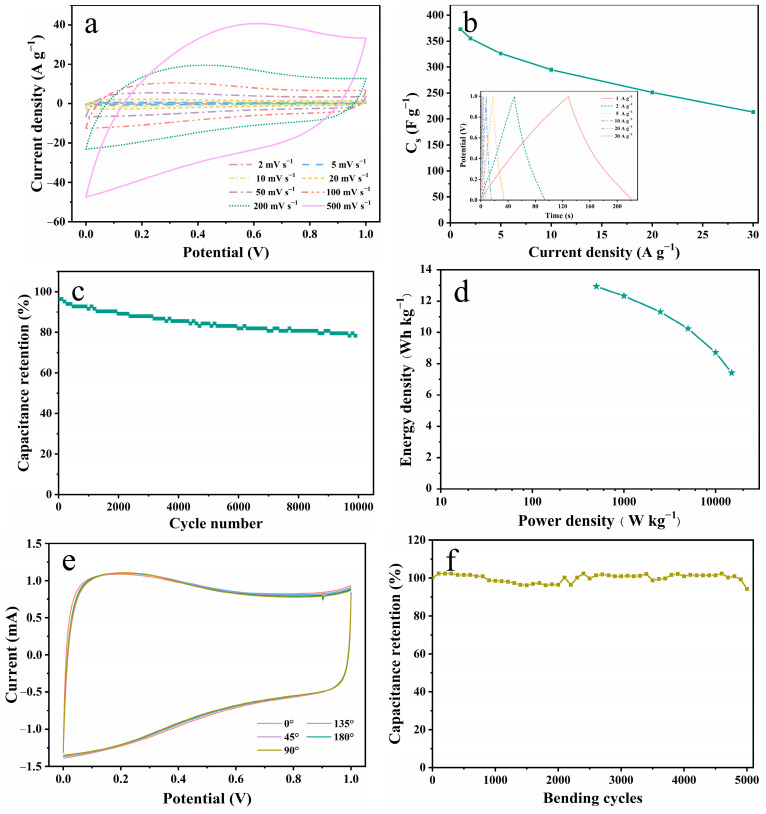
Electrochemical performance and flexibility tests of TW/rGO-based all-solid-state supercapacitor: (**a**) CV curves at different scan rates (2–500 mV s^−1^); (**b**) plots of C_s_ versus current density (inset shows GCD curves at 1–30 A g^−1^); (**c**) Ragone plot of energy density versus power density; (**d**) cycling stability at current density of 20 A g^−1^; (**e**) CV curves at 10 mV s^−1^ for different bending angles; and (**f**) capacitance retention of supercapacitor after mechanical folding cycles.

The prepared TW/rGO hydrogel exhibited good mechanical strength, a porous structure, and high specific capacitance, making it suitable for potential applications in flexible supercapacitors. In this study, a flexible all-solid-state supercapacitor based on TW/rGO hydrogel electrodes was constructed using a cellulose gel electrolyte to examine its electrochemical performance and flexibility (Figure 5). Figure 5a illustrates the CV curves of the supercapacitor at scanning rates ranging from 2 to 500 mV s^−1^. Clearly discernible redox peaks are evident in CV curves at different scanning rates, which can be attributed to pseudocapacitance peaks in rGO and TW. In addition, even at a high scanning rate of 500 mV s^−1^, the CV curves exhibits no significant distortion, indicating the excellent rate performance of the TW/rGO-based supercapacitor. The GCD curves of the device at various current densities display a relatively symmetrical triangular shape (Figure 5b), indicating high redox reversibility. Additionally, the device achieved a specific capacitance of 372.8 F g^−1^ at 1 A g^−1^ and a rate performance of 57.3% at current densities from 1 to 30 A g^−1^, demonstrating superior specific capacitance values and rate performance (Figure 5b). The outstanding specific capacitance value of the TW/rGO-based solid state supercapacitor is comparable to or even superior to other supercapacitors based on biomolecules [19,27,30,34], and even superior to other supercapacitors’ biomass-derived porous carbon [5,35,36,37,38] electrodes documented in the existing literature (detailed in Table S6). The Nyquist plot (Figure S5) shows that the supercapacitor exhibits a small semicircular and an equivalent series resistance of approximately 1.8 Ω, suggesting favorable electrolyte diffusion behavior. Figure 5c depicts the cyclic stability curve of the flexible supercapacitor, which demonstrates a capacitance retention of 78.8% after 10,000 charge–discharge cycles. The decrease in capacitance was primarily attributed to the comparatively limited reversibility of the redox reaction of oxygen-containing functional groups at a high current density. Figure 5d illustrates the energy density and power density profiles of the supercapacitor, manifesting an energy density of 12.9 Wh kg^−1^ at a power density of 500 W kg^−1^. Even when the power density was elevated to 15,000 W kg^−1^, the energy density still held at 7.4 Wh kg^−1^. These achieved values surpass those reported for the majority of previously studied biomass-based supercapacitors (Table S6), such as porous carbon derived from tea waste [36,37], L-glutamic acid functionalized nanocellulose/reduced graphene oxide [30], wood-derived carbon aerogel [39], porous carbons derived from fallen leaves [38], and so on. Furthermore, no significant changes were observed in CV tests when the device was bent to different angles ranging from 0 to 180° (Figure 5e), highlighting the outstanding flexibility and stability of the solid-state supercapacitor [27]. Furthermore, upon the accomplishment of 5000 bending cycles, the supercapacitor sustained approximately 93.4% of its initial specific capacitance at a maximum angle of 180° (Figure 5f). Thus, this TW/rGO-based flexible all-solid-state supercapacitor is regarded as a potential energy storage device for flexible wearable electronic devices.

## 4. Conclusions

A simple yet efficient strategy was proposed to directly convert tea waste into TW/rGO hydrogel electrodes with high specific capacitance and rate performance via a one-step hydrothermal method. The strong physical and chemical interactions between TW and rGO endowed the TW/rGO hydrogel with a robust 3D porous structure, thereby facilitating efficient ion transport and achieving superior rate performance. The redox-active biomolecule in TW can provide additional pseudocapacitance, significantly enhancing the electrochemical performance of TW/rGO. The all-solid-state supercapacitor assembled with TW/rGO hydrogel demonstrated superior specific capacitance (372.8 F g^−1^), competitive energy density (12.9 Wh kg^−1^), outstanding rate performance, and excellent flexibility, indicating its potential for practical applications in flexible energy storage devices. This approach of directly converting biomass waste into valuable energy is simple and efficient, holds the potential for large-scale production, and is of considerable significance in addressing environmental issues and promoting sustainable development.

## Data Availability

Data are contained within this article or the Supplementary Materials.

## Supplementary Materials

The following supporting information can be downloaded at: https://www.mdpi.com/article/10.3390/polym16223170/s1, Preparation of cellulose hydrogel; photographs of TW/rGO and rGO hydrogel (Figure S1); compressive stress-strain curves and comparison of compressive strength of TW/rGO and rGO hydrogel (Figure S2); electrochemical performance of TW/rGO hydrogels prepared at different hydrothermal temperatures (Figure S3); electrochemical performance of TW/rGO hydrogels prepared at different mass ratios of GO/TW (Figure S4); Nyquist plot of supercapacitor (Figure S5); dates of specific surface area and pore distribution (Table S1); additional dates of XRD (Table S2); relative element contents of rGO and TW/rGO (Table S3); relative content (at.%) of C1s species for rGO and TW/rGO (Table S4); capacitive performances of reported electrodes based on biomass in three-electrode system (Table S5); and capacitive performances of reported supercapacitors based on biomass (Table S6).
