# Supplementary material for: Robust Brewed Tea Waste/Reduced Graphene Oxide Hydrogel for High Performance Flexible Supercapacitors
Source: Polymers (Basel). 2024 Nov 14;16(22):3170. doi: 10.3390/polym16223170 (PMC11598633; doi:10.3390/polym16223170)
Supplement: Supplementary file 1 [file polymers-16-03170-s001.zip › polymers-3294945-supplementary.pdf]

Supporting Information

Robust Brewed Tea Waste/Reduced Graphene Oxide Hydrogel for High Performance Flexible Supercapacitors

Dan Wu [1,2], Jiajia Zhou [1], Wuqiang Deng [1], Guowen He [1,2,*] and Zheng Liu [1,2,*]

1 College of Materials and Chemical Engineering, Hunan City University, Yiyang 413000, China; wudanwd@hnu.edu.cn (D.W.); 15616298559@163.com (J.Z.); 19958821050@163.com (W.D.)
2 Key Laboratory of Low Carbon and Environmental Functional Materials of College of Hunan Province, Yiyang, 413000, China.
* Correspondence: heguowen@hncu.edu.cn (G.H.); liuzheng@hncu.edu.cn (Z.L.)

Preparation of cellulose hydrogel

Firstly, microcrystalline cellulose (600 mg) was added to 9.7 g precooled sodium hydroxide/urea/water (1 : 1.7 : 11, wt %) mixture under vigorous stirring to obtain a homogeneous viscous dispersion. The as-prepared viscous dispersion was poured into glass molds. Then 0.5 mol L^{-1} H_2SO_4 aqueous solution was slowly added to the viscous dispersion until the white cellulose hydrogel was formed. The obtained cellulose hydrogel was washed with excess water to remove residual chemicals before use.

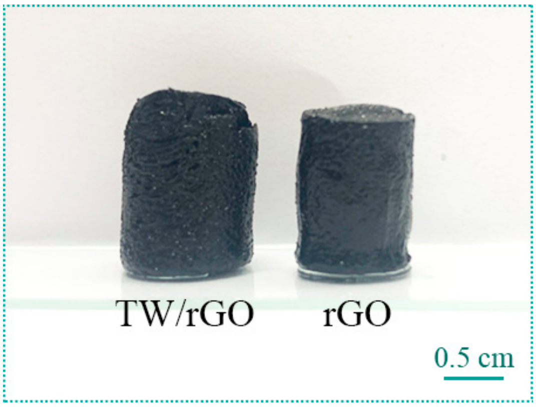

Figure S1. The photograph of TW/rGO and rGO hydrogel.

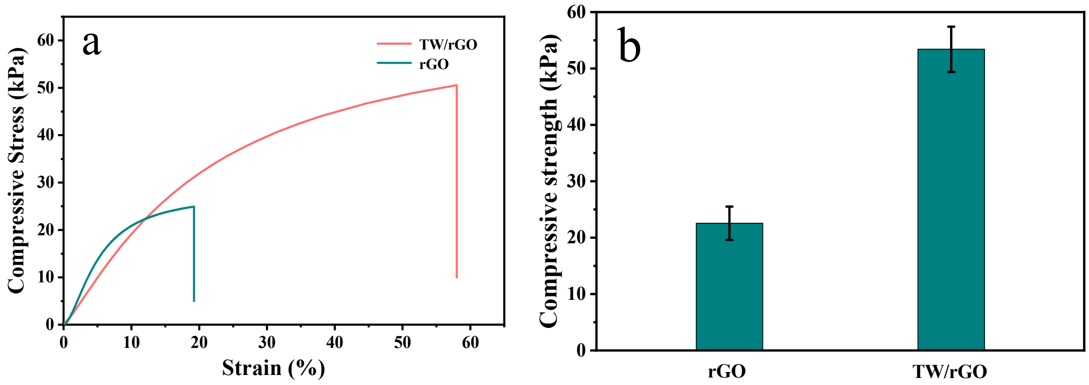

Figure S2. (a) The compressive stress-strain curves and (b) comparison of compressive strength of TW/rGO and rGO hydrogel.

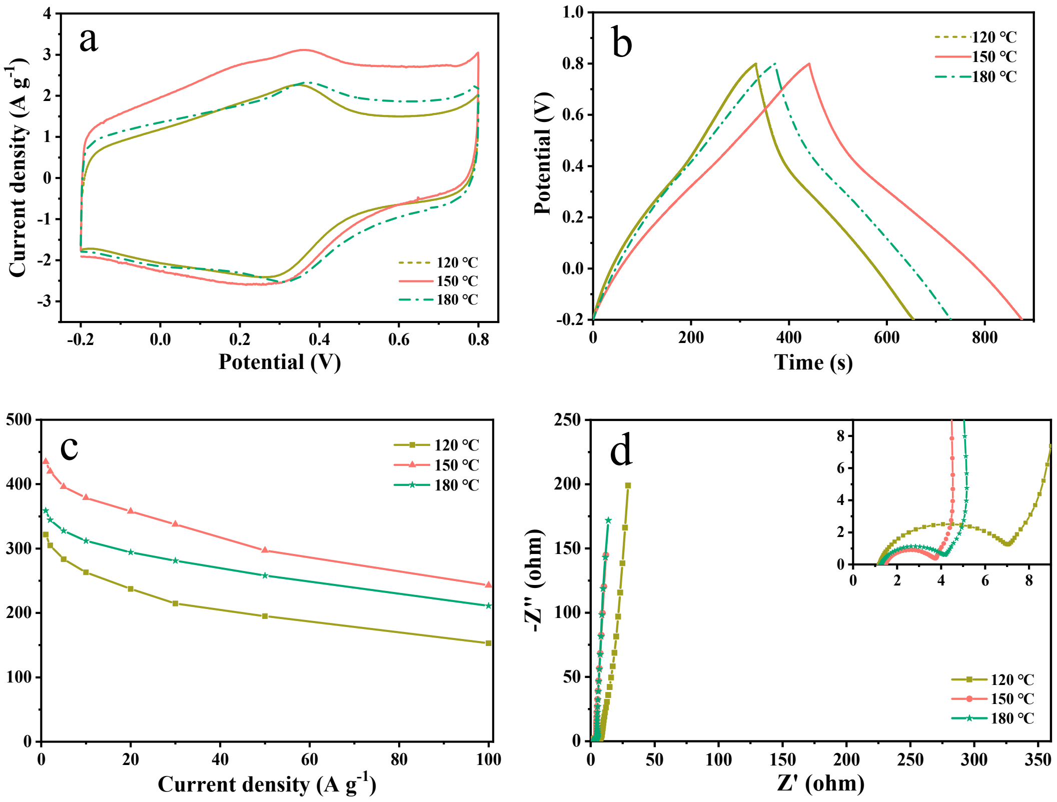

Figure S3. The electrochemical performance of TW/rGO hydrogels prepared at different hydrothermal temperatures: (a) CV curves at a scan rate of 5 mV s^{-1}, (b) GCD curves at a current density of 1 A g^{-1}, (c) Plots of C_m *versus* current density, (d) Nyquist plots.

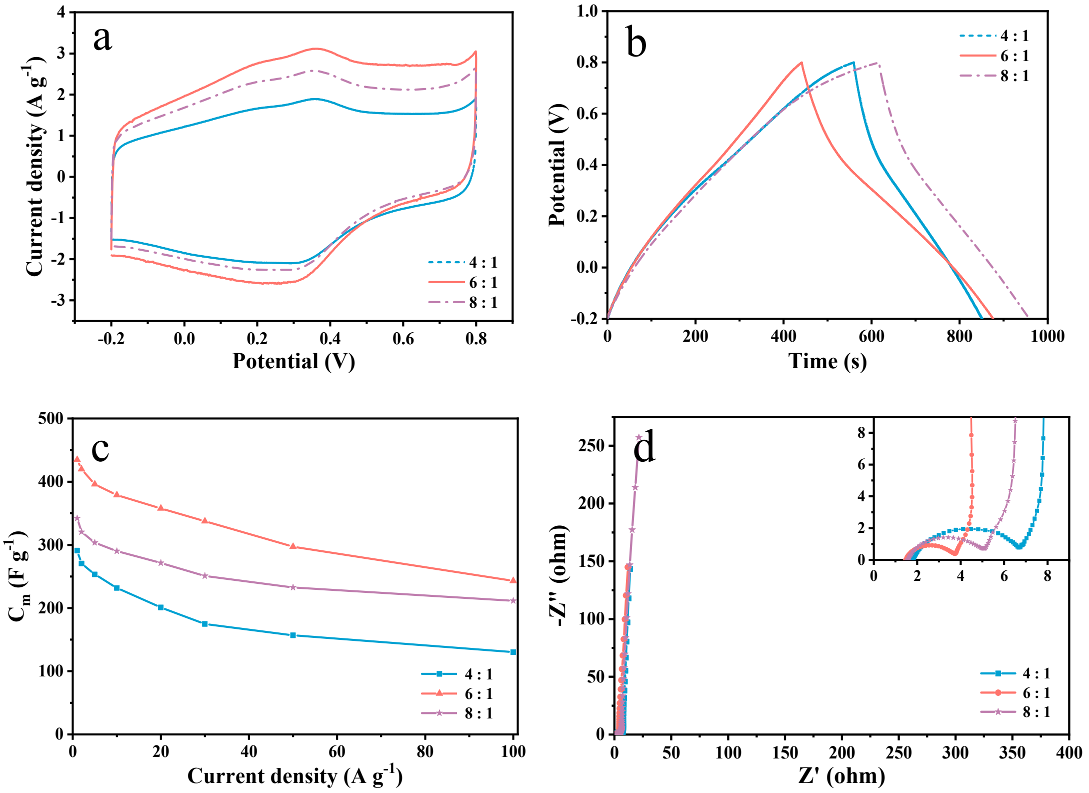

Figure S4. The electrochemical performance of TW/rGO hydrogels prepared with different mass ratio of GO to TW: (a) CV curves at a scan rate of 5 mV s^{-1}, (b) GCD curves at a current density of 1 A g^{-1}, (c) Plots of C_m *versus* current density, (d) Nyquist plots.

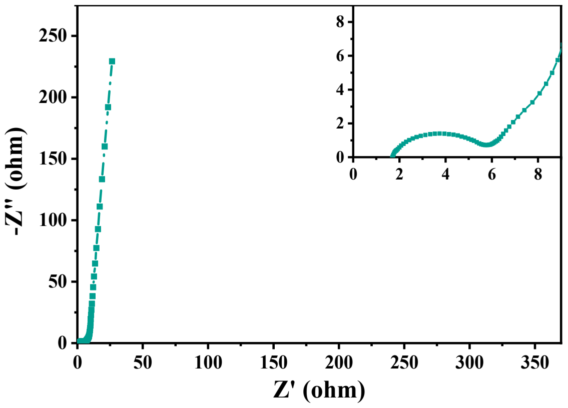

Figure S5. Nyquist plot of TW/rGO-based flexible all-solid-state supercapacitor.

Table S1. The data of specific surface area and pore distribution for rGO and TW/rGO.

Samples	BET surface area (m^2g^{-1})			Total pore Volume (cm^3g^{-1})	Average pore size (nm)
	Total	S_{micro}	S_{meso}		
rGO	66.2	0	66.2	0.19	11.8
TW/rGO	80.5	0	80.5	0.27	13.3

Table S2. The degree (2θ), d-spacing (d), and half peak width (FWHM) of rGO and TW/rGO.

Samples	2θ (°)	d (Å)	FWHM (°)
rGO	23.7	3.75	3.89
	43.2	2.09	1.68
TW/rGO	21.5	4.13	4.37
	42.8	2.11	1.26

Table S3. The relative element contents (at.%) of rGO and TW/rGO.

Samples	C	O
rGO	85.8	14.2
TW/rGO	82.3	17.7

Table S4. The relative content (at.%) of C 1s species for rGO and TW/rGO.

Samples	C=C/C-C	C-O/C-N	C=O	O-C=O
Binding energy (eV)	284.8	286.2	288.2	290.0
rGO	51.3	26.5	9.3	12.9
TW/rGO	52.2	29.3	11.5	7.0

Table S5. Capacitive performances of reported electrodes based on biomass in three-electrode system.

No.	Electrode materials	Current density	Capacitance ($F g^{-1}$)	Rate performance	Reference
1	TW/rGO	1 A g^{-1}	434.7	56% (1~100A g^{-1})	This work
2	Porous carbon derived from tea waste	0.5 A g^{-1}	170	77.6% (0.5~10A g^{-1})	[1]
3	Porous carbon derived from tea waste	1 A g^{-1}	482.1	81.3% (1~10A g^{-1})	[2]
4	GO/BLs/CDs	5 A g^{-1}	235	73.5% (0.5-5 A g^{-1})	[3]
5	Juglone/ CNTs/BC	0.5 A g^{-1}	461.8	60.6% (0.5-20 A g^{-1})	[4]
6	LS-GHs	1 A g^{-1}	432	81% (1-20 A g^{-1})	[5]
7	HCNS-RH	0.2 A g^{-1}	225	49.8% (0.2-100 A g^{-1})	[6]
8	PANI/LG	0.5 A g^{-1}	485.3	58.6% (0.5-30 A g^{-1})	[7]
9	Rhein/porous lignin-based graphitic carbon	1 A g^{-1}	250.2	46.4% (1-20 A g^{-1})	[8]
10	Porous graphitic biomass carbon from bamboo char	0.5 A g^{-1}	222	51.8% (0.5-20 A g^{-1})	[9]
11	Cornstalk-based biomass porous carbon	0.2 A g^{-1}	350.4	72.8% (0.2-20 A g^{-1})	[10]
12	Activated carbon from hazelnut shells	0.2 A g^{-1}	320.9	46.7% (0.2-30 A g^{-1})	[11]
13	Activated carbon from olive tree pruning residue	0.5 A g^{-1}	410	44% (0.5-30 A g^{-1})	[12]
14	Wood-derived carbon aerogel	0.5 A g^{-1}	260	66% (0.5~10A g^{-1})	[13]
15	Porous carbon derived from Gardenia jasminoides Ellis flowers	0.5 A g^{-1}	255	68.7% (0.5-20 A g^{-1})	[14]
16	Porous carbon derived from tobacco rods	0.5 A g^{-1}	286.6	74% (0.5-30 A g^{-1})	[15]

Table S6. Capacitive performances of reported supercapacitors based on biomass electrode materials.

No.	Electrode materials	Capacitance (F g^{-1})	Rate performance	Energy density (Wh kg^{-1})	Cycling stability	Reference
1	TW/rGO	372.8 (1 A g^{-1})	57.3% (1-30 A g^{-1})	12.9 (500 W kg^{-1})	78.8% (10000)	This work
2	Porous carbon derived from tea waste	41 (0.2 A g^{-1})	32.9% (0.2-10 A g^{-1})	8.2 (118.5 W kg^{-1})	101.4% (20000)	[1]
3	Tea waste derived microporous active carbon	167 (1 A g^{-1})	81.4% (1-30 A g^{-1})		96.7% (16000)	[16]
4	GO/BLs/CDs	94 (0.5 A g^{-1})	89.4% (0.5-2 A g^{-1})	13.4 (150 W kg^{-1})	93.5% (10000)	[3]
5	LS-GHs	408 (1 A g^{-1})	75.4% (1-20 A g^{-1})	13.8 (500 W kg^{-1})	84% (10000)	[5]
6	Lignin reduced GO aerogels	331 (1 A g^{-1})	80% (1-10 A g^{-1})		3.4% (10000)	[17]
7	L-glutamic acid functionalized nanocellulose/reduced graphene oxide (GNGH-15)	282 (0.3 A g^{-1})	79% (0.3-10 A g^{-1})	9.8 (74.9 W kg^{-1})	no decay (10000)	[18]
8	ARS-"power paper" electrode	435 (0.5 A g^{-1})		8.9 (459 W kg^{-1})		[19]
9	Wood-derived carbon aerogel	62.4 (0.5 A g^{-1})	58.5% (0.5-4.0 A g^{-1})	8.6 (250 W kg^{-1})	88.5% (5000)	[13]
10	Porous graphitic biomass carbon from bamboo char	48.1 (0.2 A g^{-1})	49.9% (0.2-20 A g^{-1})	6.68 (100.2 W kg^{-1})	84% (5000)	[9]
11	Cornstalk-based biomass porous carbon	308 (0.2 A g^{-1})	70% (0.2-20 A g^{-1})	10 (249.9 W kg^{-1})	99.8% (10000)	[10]
12	Porous carbon derived from Gardenia jasminoides Ellis flowers	153.7 (0.5 A g^{-1})	51.3% (0.5-10 A g^{-1})	5.2 (246.9 W kg^{-1})		[14]
13	Porous carbon derived from tobacco rods	54.4 (0.5 A g^{-1})	80.5% (0.5-15 A g^{-1})	~7.5 (0.5 A g^{-1})	92% (10000)	[15]
14	Sodium lignosulfonate derived hierarchical porous carbons	247 (0.05 A g^{-1})	42% (0.05-20 A g^{-1})	8.4 (151.4 W kg^{-1})	92% (10000)	[20]

15	porous carbons derived from fallen leaves	273 (0.5 A g^{-1})	86.9% (0.5-100 A g^{-1})	7.4 (151.4 W kg^{-1})	[21]
16	Fish skin-derived microporous carbon	374 (0.1 A g^{-1})	48.9% (0.1-50 A g^{-1})		[22]

References:

1. Ma, Q.; Xi, H.; Cui, F.; Zhang, J.; Chen, P.; Cui, T. Self-templating synthesis of hierarchical porous carbon with multi-heteroatom co-doping from tea waste for high-performance supercapacitor. *J. Energy Storage* **2022,** *45*, 103509.
2. Zhang, P.; Wang, W.; Kou, Z.; Wang, B.; Zhong, X. Low-cost and advanced symmetry supercapacitors based on three-dimensional tea waste of porous carbon nanosheets. *Mater. Technol.* **2020,** *36*, 1-10.
3. Wu, Q.; Jiang, C.; Zhang, S.; Yu, S.; Huang, L. Self-assembly of biomass-based hybrid hydrogel electrode for an additive-free flexible supercapacitor. *J. Mater. Chem. A* **2022,** *10*, 16853-16865.
4. Fang, D.; Zhou, J.; Sheng, L.; Tang, W.; Tang, J. Juglone bonded carbon nanotubes interweaving cellulose nanofibers as self-standing membrane electrodes for flexible high energy supercapacitors. *Chem. Eng. J.* **2020,** *396*, 125325.
5. Li, F.; Wang, X.; Sun, R. A metal-free and flexible supercapacitor based on redox-active lignosulfonate functionalized graphene hydrogels. *J. Mater. Chem. A* **2017,** *5*, 20643-20650.
6. He, D.; Gao, Y.; Wang, Z.; Yao, Y.; Wu, L.; Zhang, J.; Huang, Z. H.; Wang, M. X. One-step green fabrication of hierarchically porous hollow carbon nanospheres (HCNSs) from raw biomass: Formation mechanisms and supercapacitor applications. *J. Colloid Interface Sci.* **2021,** *581*, 238-250.
7. Wang, L.; Li, X.; Xu, H.; Wang, G. Construction of polyaniline/lignin composite with interpenetrating fibrous networks and its improved electrochemical capacitance performances. *Synth. Met.* **2019,** *249*, 40-46.
8. Wang, T.; Hu, S.; Yu, W.; Hu, Y.; Yan, S.; Wang, M.; Zhao, W.; Xu, J.; Zhang, J. Biologically inspired small herbal biomolecules and biomass carbon for

high-performance supercapacitors. *ACS Appl. Energy Mater.* **2023,** *6*, 2347-2357.

9. Gong, Y.; Li, D.; Luo, C.; Fu, Q.; Pan, C. Highly porous graphitic biomass carbon as advanced electrode materials for supercapacitors. *Green Chem.* **2017,** *19*, 4132-4140.
10. Yue, X.; Yang, H.; Cao, Y.; Jiang, L.; Li, H.; Shi, F.; Liu, J. Nitrogen-doped cornstalk-based biomass porous carbon with uniform hierarchical pores for high-performance symmetric supercapacitors. *J. Mater. Sci.* **2022,** *57*, 3645-3661.
11. Reddygunta, K. K. R.; Callander, A.; Šiller, L.; Faulds, K.; Berlouis, L.; Ivaturi, A. Sono-exfoliated graphene-like activated carbon from hazelnut shells for flexible supercapacitors. *Int. J. Energy Res.* **2022,** *46*, 16512-16537.
12. Ponce, M. F.; Mamani, A.; Jerez, F.; Castilla, J.; Ramos, P. B.; Acosta, G. G.; Sardella, M. F.; Bavio, M. A. Activated carbon from olive tree pruning residue for symmetric solid-state supercapacitor. *Energy* **2022,** *260*, 125092.
13. Chen, Z.; Zhuo, H.; Hu, Y.; Lai, H.; Liu, L.; Zhong, L.; Peng, X. Wood-derived lightweight and elastic carbon aerogel for pressure sensing and energy storage. *Adv. Funct. Mater.* **2020,** *30*, 1910292.
14. Qin, C.; Wang, S.; Wang, Z.; Ji, K.; Wang, S.; Zeng, X.; Jiang, X.; Liu, G. Hierarchical porous carbon derived from Gardenia jasminoides Ellis flowers for high performance supercapacitor. *J. Energy Storage* **2021,** *33*, 102061.
15. Zhao, Y.-Q.; Lu, M.; Tao, P.-Y.; Zhang, Y.-J.; Gong, X.-T.; Yang, Z.; Zhang, G.-Q.; Li, H.-L. Hierarchically porous and heteroatom doped carbon derived from tobacco rods for supercapacitors. *J. Power Sources* **2016,** *307*, 391-400.
16. Song, X.; Ma, X.; Li, Y.; Ding, L.; Jiang, R. Tea waste derived microporous active carbon with enhanced double-layer supercapacitor behaviors. *Appl. Surf. Sci.* **2019,** *487*, 189-197.
17. Jiang, C.; Wang, Z.; Li, J.; Sun, Z.; Zhang, Y.; Li, L.; Moon, K.-S.; Wong, C. RGO-templated lignin-derived porous carbon materials for renewable high-performance supercapacitors. *Electrochim. Acta* **2020,** *353*, 136482.
18. Zhang, Y.; Liu, K.; Ma, W.; Wang, C.; Yu, T.; Chen, J.; Fan, S. High-performance supercapacitors based on compact graphene composite hydrogels. *Electrochim.*

Acta **2024,** *476*, 143699.

19. Edberg, J.; Brooke, R.; Granberg, H.; Engquist, I.; Berggren, M. Improving the performance of paper supercapacitors using redox molecules from plants. *Adv. Sust. Syst.* **2019,** *3*, 1900050.
20. Pang, J.; Zhang, W.; Zhang, J.; Cao, G.; Han, M.; Yang, Y. Facile and sustainable synthesis of sodium lignosulfonate derived hierarchical porous carbons for supercapacitors with high volumetric energy densities. *Green Chem.* **2017,** *19*, 3916-3926.
21. He, J.; Zhang, D.; Wang, Y.; Zhang, J.; Yang, B.; Shi, H.; Wang, K.; Wang, Y. Biomass-derived porous carbons with tailored graphitization degree and pore size distribution for supercapacitors with ultra-high rate capability. *Appl. Surf. Sci.* **2020,** *515*, 146020.
22. Niu, J.; Liu, M.; Xu, F.; Zhang, Z.; Dou, M.; Wang, F. Synchronously boosting gravimetric and volumetric performance: Biomass-derived ternary-doped microporous carbon nanosheet electrodes for supercapacitors. *Carbon* **2018,** *140*, 664-672.
